# *KIDINS220* Variant Associated With Hypoplasia of the Corpus Callosum and Aqueduct Stenosis

**DOI:** 10.1002/pd.6804

**Published:** 2025-05-02

**Authors:** Kimia Ghannad‐Zadeh, Patrick Shannon, Rebekah Jobling, Elka Miller, Karen Chong, Erin Mathews, David Chitayat, Shiri Shinar

**Affiliations:** ^1^ Temerty Faculty of Medicine University of Toronto Toronto Canada; ^2^ Department of Pathology and Laboratory Medicine Mount Sinai Hospital University of Toronto Toronto Canada; ^3^ Genome Diagnostics Department of Pediatric Laboratory Medicine The Hospital for Sick Children Toronto Canada; ^4^ Division of Clinical and Metabolic Genetics Department of Pediatrics The Hospital for Sick Children University of Toronto Toronto Canada; ^5^ Department of Diagnostic and Interventional Radiology SickKids Hospital University of Toronto Toronto Canada; ^6^ The Prenatal Diagnosis and Medical Genetics Program Department of Obstetrics and Gynecology Mount Sinai Hospital University of Toronto Toronto Canada; ^7^ Ontario Fetal Center Division of Maternal Fetal Medicine Department of Obstetrics and Gynecology Mount Sinai Hospital University of Toronto Toronto Canada

## Abstract

*KIDINS220* plays a key role in neuronal survival, differentiation, and synaptic function. Abnormalities in its expression have been linked postnatally to neurodevelopmental disorders and SINO syndrome though prenatal presentations are rarely described. We report a novel de novo heterozygous *KIDINS220* variant identified prenatally associated with bilateral ventriculomegaly, abnormal anterior horns, aqueductal stenosis, and a hypoplastic corpus callosum. This is the first prenatal case of such findings in *KIDINS220*, emphasizing the value of trio WES/WGS for diagnosis and counseling.

**Summary**

- What's already known about this topic?
  ◦ Aberrant expression of *KIDINS220* has been associated postnatally with neurodevelopment abnormalities and SINO syndrome.
  ◦ Most individuals with SINO syndrome present postnatally with macrocephaly and ventriculomegaly. Prenatal presentation and neuropathological findings are rarely reported.
- What does this study add?
  ◦ We report a case with hypoplasia of the corpus callosum, aqueduct stenosis and dilation of the anterior horns secondary to a de novo likely pathogenic heterozygous variant in KIDINS220.
  ◦ To the best of our knowledge, this is the first prenatal report of these brain anomalies associated with the KIDINS220 variant.

## 1 Fetal Phenotype

**FIGURE 1:**
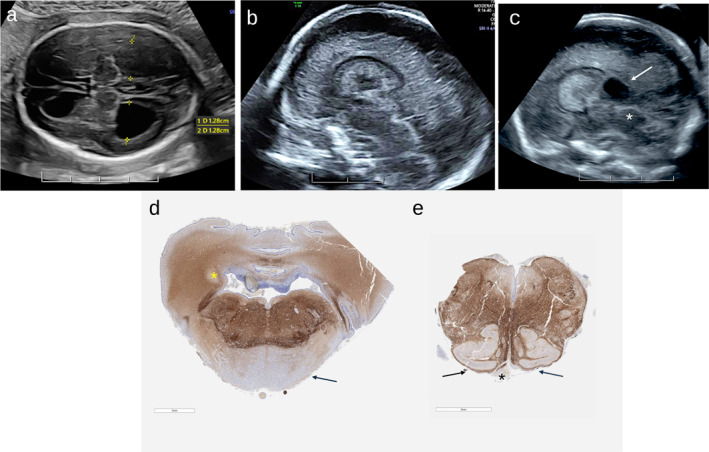
(a) Ultrasound images at 20 + 5 weeks of gestation axial plane demonstrating moderate bilateral ventriculomegaly (12.8 mm) and marked dilation of the anterior horns (arrows) as well as cortical thinning posteriorly. Midsagittal plane with diffusely hypoplastic corpus callosum. (b, c) Dilation of posterior recess of third ventricle (white arrow) secondary to aqueduct stenosis (white asterix). (d) Brainstem sections, stained with antibodies to Neurofilament Light Chain demonstrating demonstrating relatively small cerebellum, basis pontis (arrow) and small, poorly demarcated dentate nuclei (asterix). (e) Medulla with absent pyramids (asterix) and dysplastic inferior olivary nuclei (arrows).

The mother was a 33 years old primigravida and the father were of the same age. The couple was healthy and non‐consanguineous, and their family history was noncontributory. The conception was natural and resulted in dichorionic diamniotic twins with a vanishing twin in the first trimester. A detailed anatomical survey at 19 weeks showed bilateral ventriculomegaly of 14 mm and query absence of the cavum septi pellucidi (CSP). No extra CNS anomalies were seen. A neurosonogram at 20 + 5 weeks showed an appropriate gestational age male with bilateral moderate ventriculomegaly (13 mm, HP:0002119) with marked ballooning of the anterior horns and mild prominence of the third ventricle measuring 3 mm (Figure 1a). No fluid was seen in the aqueduct, raising suspicion for aqueduct stenosis (HP:0002410). There was a single choroid plexus cyst (HP:0011464). The CSP was thinned and compressed with a diffusely hypoplastic corpus callosum (HP:0002079 Figure 1b). The parieto‐occipital sulci were mildly effaced secondary to the ventriculomegaly and sulcation was otherwise age appropriate. There was bilateral thinning of the occipital parenchyma (HP:0002450, Tables 1A and 1B). Amniocentesis and MRI declined.

**TABLE 1A:** Clinical data.

Case	Parental details			Gestation at diagnosis	Phenotypes (HPO terms)	Obstetric history	Family history	Outcome
1	Maternal	Age	33	20 + 5 weeks	Mesomelia (HP:0003027) Macrocephaly (HP:0000256) Brachycephaly (HP:0000248) Frontal bossing (HP:0002007) Flat face (HP:0012368) Thickened nuchal skin fold (HP:0000474) Aplasia of the fingers (HP:0009380) Aplasia of the toes (HP:0001991) Hemivertebrae (HP:0002937) Micropenis (HP:0000054)	G3P0020 Two first trimester miscarriages	Nil	Termination of pregnancy
		Ethnicity	English					
	Paternal	Age	33					
		Ethnicity	Sepharadic‐Jewish					

**TABLE 1B:** Sequence variants in *KIDINS220*.

Gene transcript	Mode of inheritance, gene OMIM	DNA variants, predicted effects, zygosity	ClinVar ID	Highest allele frequency in a gnomAD population	Interpretation
*COL4A1*,	AD, 120130	c.1536 + 1G > A, GT donor, heterozygous (de novo)	3061119	Not present	Pathogenic PVS1 PS2 PM2
NM_001845.5					
*KIDINS220*	AD, AR, 615759, 617296, 619501	c.3899del; p.Pro1300Argfs*34, heterozygous (de novo)	3349692	Not present	Pathogenic PVS1 PS2 PM2
NM_020738.2					

## 2 Diagnostic Method

NIPT at 10 weeks gestation showed low risk for Trisomy 21, 13, 18, and sex chromosome aneuploidy in dizygotic male twins.

Early detailed ultrasound at 12 + 1 weeks showed the demise of one of the twins and the NT of the remaining twin was 1.1 mm.

Microarray analysis done on fetal banked DNA (fDNA) obtained at autopsy using an Illumina CytoSNP‐850K array showed a male fetus with no copy number gains or losses greater than 10 Kb across the genome.

## 3 Diagnostic Results and Interpretation

Trio whole‐exome sequencing performed on fDNA showed two likely pathogenic de novo sequence variants, one in *KIDINS220* (c.3899del; p. Pro1300Argfs * 34) predicted to result in a frameshift, and a sequence variant in *COL4A1* (c.1536 + 1G > A, GT donor) predicted to disrupt the GT donor site and interfere with normal splicing (Tables 1A and 1B). The couple was counseled that since these variants had likely arisen de novo, the empiric recurrence risk was approximately 1% due to the possibility of germline mosaicism.

## 4 Pregnancy Outcome and Autopsy Findings

The couple decided to terminate the pregnancy and the patient underwent an induction of labor and uncomplicated vaginal delivery at 21 + 1 weeks. Autopsy showed mainly brain anomalies including hypoplasia of the corpus callosum, mild aqueduct stenosis, severe hypoplasia of the corticofugal tracts and hypoplasia of the rhombic lip derivatives (Figure 1c,d).

## 5 Discussion

*KIDINS220* codes for a protein preferentially expressed in the nervous system that controls neuronal cell survival, axonal and dendritic differentiation, and synaptic plasticity. Aberrant expressions of *KIDINS220* have been associated with various neuropsychiatric and neurodegenerative diseases. Early termination and heterozygous loss‐of‐function variants in the last two exons of this gene are associated with pastic paraplegia, intellectual disability, nystagmus, and obesity (SINO syndrome) [1]. Other reported functional abnormalities include behavioral abnormalities, autism spectrum disorder, and attention deficit/hyperactivity disorder.

The effects of *KIDIND220* variants on neurodevelopment are further characterized in mouse models. *KIDINS220* knockout mice are not viable, with extensive deficits in dendritic growth, high rates of neuronal cell death, and significant ventriculomegaly [2]. Heterozygous *KIDINS220* ^+/−^ mice are viable and do not show any major behavioral phenotype [2].

Biallelic KIDS220 likely pathogenic/pathogenic (LP/P) variants have been reported prenatally in three cases with the dominant early prenatal finding of ventriculomegaly [3, 4, 5]. In a recent report, Miremberg et al. reported a novel heterozygous variant in *KIDINS220* presenting at 17 weeks with corpus callosum and brain stem dysgenesis, cortical malformation and hypertelorism [6]. The prenatal findings in our case are unique, consisting of ventriculomegaly, hypoplasia of the corpus callosum, and aqueduct stenosis on US and agenesis of the corticofugal tracts and hypoplasia of the rhombic lip derivatives on histopathology. These findings have yet to be described prenatally for this variant.

The variant reported here was heterozygous and resulted in frameshift and premature protein termination in exon 29 of the *KIDINS220* gene. To the best of our knowledge, this variant has not been reported in the literature or in large population databases. However, due to its location in exon 29, it is expected to be pathogenic for autosomal dominant and recessive *KIDINS220* associated disorders. The fetus reported here was also heterozygous for a de novo, likely pathogenic variant in *COL4A1*. In our reported case, there were no anomalies observed in neuromuscular or cardiac development, and there were no signs of intracranial hemorrhage, phenotypes characteristic of pathogenic *COL4A1* variants.

In summary, we report a new LP de novo variant in *KIDINS220* associated with aqueduct stenosis that would have likely resulted in SINO syndrome had the pregnancy continued. Our case highlights the importance of dedicated neurosonographic assessment of fetuses with ventriculomegaly as well as the importance of trio WES/WGS for accurate diagnosis and prenatal counseling.

## Ethics Statement

The authors have nothing to report.

## Consent

The parents consented for publication.

## Conflicts of Interest

The authors declare no conflicts of interest.

## Data Availability

The data supporting the findings of this study are not publicly available due to privacy restrictions. However, they are available from the corresponding author upon reasonable request and with appropriate permissions, where applicable.
